# Notch signaling increases PPARγ protein stability and enhances lipid uptake through AKT in IL‐4‐stimulated THP‐1 and primary human macrophages

**DOI:** 10.1002/2211-5463.12858

**Published:** 2020-04-26

**Authors:** Naunpun Sangphech, Pornlapat Keawvilai, Tanapat Palaga

**Affiliations:** ^1^ Inter‐disciplinary Graduate Program in Medical Microbiology Graduate School Chulalongkorn University Bangkok Thailand; ^2^ Center of Excellence in Immunology and Immune‐mediated Diseases Chulalongkorn University Bangkok Thailand; ^3^ Graduate Program in Biotechnology Faculty of Science Chulalongkorn University Bangkok Thailand; ^4^ Department of Microbiology Faculty of Science Chulalongkorn University Bangkok Thailand

**Keywords:** AKT, human monocyte‐derived macrophage, IL‐4, Notch signaling, PPARγ

## Abstract

Notch signaling and nuclear receptor PPARγ are involved in macrophage polarization, but cross talk between them has not been reported in macrophages. In this study, the effect of Notch signaling on PPARγ in IL‐4‐stimulated human macrophages (M(IL‐4)) was investigated using THP‐1‐derived macrophages and human monocyte‐derived macrophages as models. Human M(IL‐4) increased the expression of JAGGED1 and activated Notch signaling. Overexpression of Notch1 intracellular domain (NIC1) increased PPARγ expression, while inhibiting Notch signaling decreased PPARγ levels in M(IL‐4). NIC1 overexpression in THP‐1‐derived macrophages increased PPARγ protein stability by delaying its proteasome‐mediated degradation, but did not affect its mRNA. Phosphorylation of AKT was enhanced in NIC1‐overexpressing cells, and a specific AKT inhibitor reduced the level of PPARγ. NIC1‐overexpressing THP‐1 cells exhibited increased CD36 levels via activation of PPARγ, resulting in enhanced intracellular lipid accumulation. In summary, this study provides evidence linking Notch signaling and PPARγ via AKT in M(IL‐4).

## Graphical abstract

Notch signaling is known to regulate activation of pro‐inflammatory macrophages. Here, we report that IL‐4‐stimulated human macrophages (M(IL‐4)) promptly activate Notch signaling via JAGGED1. This activation is accompanied by increased PPARγ, a key transcription factor for M(IL‐4). Cross talk between Notch signaling and AKT/ERK pathways was observed. Interfering with Notch signaling affects PPARγ level and its biological functions.

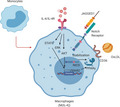

**Abbreviations**
DN: dominant negative
HMDM: human monocyte‐derived macrophage
LPS: lipopolysaccharide
MAML: Mastermind‐like 1
NIC1: Notch1 intracellular domain
PPAR: peroxisome proliferator‐activated receptor

Macrophages have multifaceted functions in the immune system depending on their microenvironments and the stimuli they receive [1, 2]. In an *in vitro* system, macrophages are polarized to two opposing phenotypes: the pro‐inflammatory [such as LPS‐stimulated (M(LPS)] and pro‐healing [such as M(IL‐4)] phenotypes. Both types play important roles in tissue homeostasis and the pathogenesis of many diseases [2]. The reversal of the pro‐inflammatory to the pro‐healing phenotype reduced the plaque size and resulted in a good disease prognosis for atherosclerosis [3]. However, tumor‐associated macrophages showed a pro‐healing‐like phenotype and supported tumor progression and metastasis [4]. Consequently, macrophage activation is a double‐edged sword, and it is expected that controlling its activation can be an alternative therapeutic choice for such chronic conditions [3, 5].

Interleukin‐4 (IL‐4) is a well‐known cytokine that activates macrophages and induces pro‐healing phenotypes [6]. IL‐4/IL‐4R signaling activates STAT6 and AKT1, leading to a second wave of the activation of transcription factors, such as PPARγ, a key transcription factor of M(IL‐4) [7, 8, 9, 10]. M(IL‐4) upregulates a set of genes involved in anti‐inflammation, lipid metabolism, apoptotic cell clearance, and cellular metabolism [1, 11, 12, 13].

PPARγ is a ligand‐dependent nuclear hormone receptor [14]. The target genes of PPARγ in macrophages include *CD36*, *LPL* (lipoprotein lipase), and *FABP4* (fatty acid binding protein P4) [15]. The products of these genes are necessary for metabolic regulation in macrophages [14, 16]. Furthermore, PPARγ‐deficient macrophages exhibited impaired phagocytic activity to clear apoptotic cells in wounds, resulting in increased TNFα production [12].

PPARγ degradation is regulated mainly by proteasomal degradation, which is mediated through PPARγ E3 ligases [17]. MAPK/ERK‐kinase 1/2 (MEK1/2) activation directly interacts with PPARγ in the nucleus and supports its export to function in a transcriptional activity‐independent manner or its degradation by the proteasome in the cytoplasm [18]. IFNγ‐induced PPARγ phosphorylation at Ser112 by ERK1/2 targets it for ubiquitination and proteasomal degradation in adipocytes [18, 19]. In addition, PI3K/AKT and protein kinase A are required for the transcriptional activation of *PPARG*, while increased PI3K activity reduced its protein activity in adipocytes [20, 21].

The conserved Notch signaling pathway was reported to be a key factor operating during macrophage activation [22, 23]. The inhibition of Notch signaling is achieved by using γ‐secretase inhibitor to suppress the cleavage of Notch receptors [24] or the overexpression of a dominant negative form of Mastermind‐like protein (MAML), which functions as a scaffold protein for the formation of the Notch‐CSL/RBP‐Jκ complex. Notch activation is required for the activation of pro‐inflammatory macrophages [22, 25]. Forced Notch activation in macrophages with the Notch ligand DLL4 switched macrophages to a pro‐inflammatory phenotype with increased tumoricidal activity in a tumor model, suggesting a pro‐inflammatory role [26]. In contrast, it was found in a breast cancer model that CSL/RBP‐Jκ is important for the differentiation of tumor‐associated macrophages and that the deletion of *Rbpj* in murine macrophages resulted in defects in chitin‐mediated M2 differentiation [27, 28]. Therefore, the role of Notch signaling in M(IL‐4) is still controversial and requires further investigation.

The cross talk between Notch signaling and PPARγ has been reported. During keratinocyte differentiation, Jagged1 increases PPARγ expression and inhibits the physical association between NF‐κB p65, and PPARγ, possibly through Notch activation. This association caused keratinocytes to undergo terminal differentiation [29]. In 3T3‐L1 cells, a preadipocyte cell line, Notch1 upregulates PPARγ and PPARδ, which are necessary for adipocyte differentiation [30]. In this study, we uncovered the role of Notch signaling on the stability of PPARγ in M(IL‐4) through AKT. The impact and the mechanism of this cross talk in M(IL‐4) using human monocyte‐derived macrophage (HMDM) and THP‐1‐derived macrophages as model are presented.

## Materials and methods

### Cell culture and primary human macrophages and inhibitors

Ethics approval for the use of healthy donor blood was granted by the Institutional Review Board, Faculty of Medicine at Chulalongkorn University (IRB No. 055/60). All methods were performed in accordance with the relevant guidelines and regulations by Chulalongkorn University. Written informed consent for study participation was obtained before the samples were collected. The study methodologies conformed to the standards set by the Declaration of Helsinki. To generate HMDMs, CD14^+^ monocytes were separated from peripheral blood mononuclear cells by human CD14 MicroBeads (MACS Miltenyi Biotec, Bergisch Gladbach, Germany). CD14^+^ monocytes were maintained in complete medium [iMDM media supplemented with 5% human serum and antibiotics (HyClone, Cramlington, UK) for 7 days supplemented with M‐CSF (20 ng·mL^−1^; BioLegend, San Diego, CA, USA)]. THP‐1, a human monocytic leukemia cell line (JCRB0112, National Institutes of Biomedical Innovation, Health and Nutrition Japanese Collection of Research Bioresources, Japan), was cultured in RPMI‐1640. To generate THP‐1‐derived macrophages, cells were treated with phorbol 12‐myristate 13‐acetate (PMA; Calbiochem, San Diego, CA, USA) (5 ng·mL^−1^) for 2 days to differentiate the cells from monocytes to macrophages. All specific inhibitors [LY294002, DAPT (Merck Millipore, Burlington, MA, USA), U0126 (Cell Signaling Technology, Danvers, MA, USA) and T0070907 (Selleckchem, Houston, TX, USA)], were dissolved in DMSO.

### Retroviral and lentiviral transduction

The retroviral plasmid vectors for DNMAML (MSCV‐Mam(12–74)‐EGFP) and NIC1(MSCV‐GFP‐Myc‐NIC1) were a kind gift from W. Pear (University of Pennsylvania, USA) and B. A. Osborne (University of Massachusetts Amherst, USA), respectively. A control empty vector, MSCV‐IRES‐GFP (plasmid 20672), was obtained from Addgene (Watertown, MA, USA). The retroviral vectors and the packaging construct pCL‐Ampho (Imagenex, Port Coquitlam, British Columbia, Canada) were cotransfected into 293T cells using the FuGene^®^ HD transfection reagent (Roche, Indianapolis, IN, USA) according to the manufacturer's instructions. The transfection efficiency was confirmed by fluorescence microscopy and flow cytometry.

### Western blot analysis

The primary antibodies used are as follows: anti‐cleaved Notch1 Ab (Val1744), anti‐PPARγ Ab, anti‐phosphor‐STAT6 Ab, anti‐total‐STAT6 Ab, anti‐phosphor‐AKT Ab, anti‐total‐AKT Ab, anti‐phosphor‐ERK1/2 Ab and anti‐total‐ERK1/2 (all were purchased from Cell Signaling Technology), anti‐Notch1 Ab (Santa Cruz Biotech, Dallas, TX, USA), and anti‐β‐actin Ab (Merck Millipore). The secondary antibodies used in this study were as follows: horseradish peroxidase‐conjugated sheep anti‐mouse IgG Ab (GE Healthcare, Chicago, IL, USA) and horseradish peroxidase‐conjugated goat anti‐rabbit IgG Ab (Cell Signaling Technology). The signal was detected using the Amersham Hyperfilm™ ECL chemiluminescent detection method (Amersham Bioscience, Piscataway, NJ, USA).

### Real‐time quantitative PCR

Cells were treated as indicated, and the total RNA was extracted using TRIzol^®^ (Invitrogen, Waltham, MA, USA). Quantitative PCR (qPCR) was performed using 0.1–1 µg of RNA and the iQ™SYBR^®^Green SuperMix (Bio‐Rad, Hercules, CA, USA) following the manufacturer's protocol, and qPCR was performed using Bio‐Rad CFX Connect Real Time System (Bio‐Rad). The relative mRNA expression levels were calculated and analyzed as previously described [31].

### JAGGED1 blockade using neutralizing antibody

THP‐1‐derived macrophages were treated with neutralizing antibody against JAGGED1 (R&D Systems^®^, Minneapolis, ‎MN, USA) (10 µg·mL^−1^) or isotype control antibody (R&D Systems^®^) (10 µg·mL^−1^) for 1 h. Cells were stimulated with IL‐4 (20 ng·mL^−1^) for 3 h. RNA was collected and investigated mRNA expression by real‐time quantitative PCR.

### Flow cytometry

Cells were harvested, and the FC receptor was blocked with human serum. Anti‐IL‐4Rα‐PE Ab (BioLegend), anti‐CD36‐PE Ab (ImmunoTools, Friesoythe, Germany), or dead‐live dye 7‐AAD (BD™ PharMingen, Franklin Lakes, NJ, USA) was used for staining. Cells were analyzed by flow cytometry (FC500; Beckman Coulter, Brea, CA, USA). The acquired data were analyzed using flowjo data analysis software (Tree Star, Inc., Ashland, OR, USA).

### mRNA stability assay

Cells were stimulated with IL‐4 (20 ng·mL^−1^) for 3 h prior to actinomycin D (ActD, 1 μg·mL^−1^) (Merck, Darmstadt, Germany) treatment. Total RNA was collected at the indicated times and subjected to qPCR. The mRNA half‐life was calculated using the equation: *T*1/2 = ln2/*k*, where *k* is the constant value of mRNA degradation [32].

### Protein degradation and half‐life

Cells were pretreated with MG132 (Calbiochem) (1 μm) before stimulation with IL‐4 (20 ng·mL^−1^) for 4 h. Cells were stimulated with IL‐4 (20 ng·mL^−1^) for 4 h prior to cycloheximide (CHX, 1 μg·mL^−1^; Sigma, St. Louis, MO, USA) treatment. Protein lysates were subjected to western blot. The band density of proteins was quantitated using imagej software (NIH, Bethesda, MD, USA).

### Lipid staining

Cells were stimulated as indicated, and fixed cells were stained with Oil Red O solution for 10 min. Excessive dye was washed off with water four times. Cells were visualized under an inverted microscope (Olympus, Olympus Corporation, Japan), the staining was quantified by lipid elusion with 100% isopropanol, and the OD at 492 nm was measured.

### Statistical analysis

All assays were performed in at least three independent experiments; for example, each experiment was repeated at least three times when using cell line, and each experiment was done using at least duplicate samples. All statistical analyses were performed using graphpad prism software (San Diego, CA, USA); the statistical significance was determined using two‐way ANOVA, one‐way ANOVA, or unpaired *t*‐test. A *P*‐value of less than 0.05 was considered significant.

## Results

### IL‐4 activates Notch signaling via JAGGED1 in THP‐1‐derived macrophages and HMDMs

**Fig. 1:**
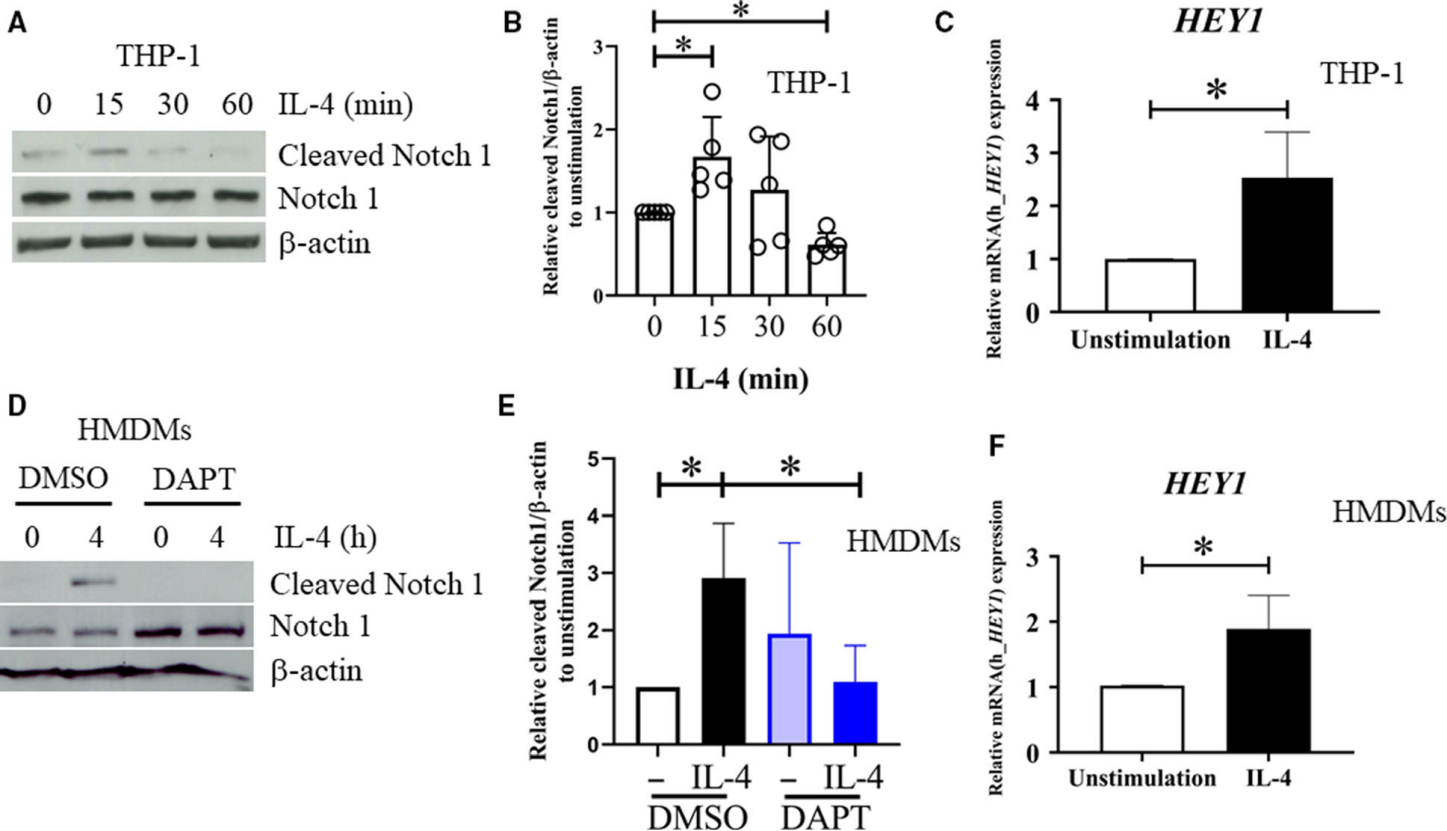
Activation of Notch signaling in M(IL‐4). THP‐1‐derived macrophages were stimulated with IL‐4 (20 ng·mL^−1^) for the indicated times. (A) Cleaved Notch1 and Notch1 were detected by western blotting. (B) The relative densitometric intensity of cleaved Notch1 was normalized with β‐actin and compared to that of the unstimulation control. (C) *HEY1* mRNA expression was examined by qPCR. *β‐ACTIN* was used as a housekeeping gene by unpaired *t*‐test. The error bars represent mean ± SD. (D) HMDMs were pretreated with DAPT (50 μm) or vehicle control (DMSO) before stimulation with IL‐4 (20 ng·mL^−1^) for 4 h. Cleaved Notch1 and Notch1 were examined by western blotting. (E) The densitometric intensity of cleaved Notch1 was normalized with β‐actin and compared to that of the DMSO‐treated control. * indicates statistically significant differences at *P* < 0.05 by paired *t*‐test. The error bars represent mean ± SEM. (F) *HEY1* mRNA expression was examined by qPCR. *β‐ACTIN* was used as a housekeeping gene. The result was performed from 3 to 7 healthy donors or at five independent experiment in THP‐1‐derived macrophages. * indicates statistically significant differences at *P* < 0.05 by unpaired *t*‐test. The error bars represent mean ± SD.

To examine whether Notch signaling plays a role in M(IL‐4), the activation of Notch signaling was examined by detection of cleaved Notch1 (Val1744). A rapid appearance of cleaved Notch1 within 15 min after IL‐4 stimulation in the THP‐1‐derived macrophages was observed (Fig. 1A,B). Moreover, the mRNA level of one of the Notch target genes, *HEY1*, was also increased upon IL‐4 stimulation (Fig. 1C). Similar results were obtained from HMDMs (Fig. 1D–F). Pretreatment with γ‐secretase inhibitor (DAPT) before IL‐4 stimulation completely abrogated the appearance of cleaved Notch1 in M(IL‐4) (Fig. 1D).

**Fig. 2:**
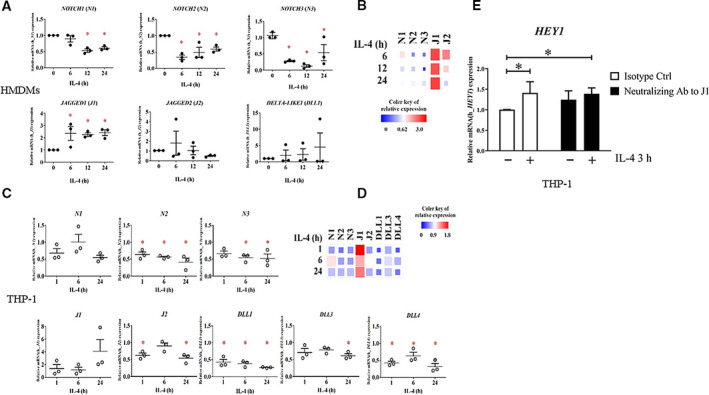
Expression of Notch ligands and receptors in M(IL‐4). (A, B) HMDMs or (C, D) THP‐1‐derived macrophages were stimulated with IL‐4 (20 ng·mL^−1^) for indicated time. Total RNA was collected to examine mRNA expression of Notch ligands and receptors by qPCR. *β‐ACTIN* was used as housekeeping gene. (B, D) Summary of relative mRNA level was shown in as heatmap. The box size correlated with the expression level. * indicates statistically significant differences when compared with unstimulated condition at *P* < 0.05 by unpaired *t*‐test. The error bars represent mean ± SEM. (E) THP‐1‐derived macrophages were treated with neutralizing antibody against JAGGED1 (J1) (10 µg·mL^−1^) for 1 h before stimulating with IL‐4 (20 ng·mL^−1^) for 3 h. *HEY1* mRNA expression was determined by qPCR. The error bars represent mean ± SD. The results are from three healthy donors or three independent experiments in THP‐1‐derived macrophages. * indicates statistically significant differences when compared with unstimulated condition at *P* < 0.05 by one‐way ANOVA.

To identify which Notch ligand(s) play a role in initiating Notch signaling upon IL‐4 stimulation, we examined mRNA expression of Notch receptors and ligands by qPCR. The mRNA expression of all Notch ligands and receptors, except *JAGGED1*, was downregulated upon IL‐4 stimulation in HMDMs and THP‐1‐derived macrophages (Fig. 2A–D). *NOTCH4* was undetectable in all experiments. Therefore, NOTCH1 and JAGGED1 were potentially involved in IL‐4‐activated Notch signaling. We tested whether JAGGED1 activates Notch signaling during IL‐4 stimulation by using neutralizing antibody against JAGGED1. JAGGED1‐neutralizing antibodies effectively reduced mRNA of *HEY1* upon IL‐4 stimulation (Fig. 2E), suggesting that JAGGED1 is the main ligand that triggers Notch signaling in M(IL‐4). Together, these results suggest that IL‐4 activates Notch signaling in human macrophages mainly via JAGGED1, which requires γ‐secretase.

### Notch signaling increases PPARγ expression but not its mRNA in M(IL‐4)

**Fig. 3:**
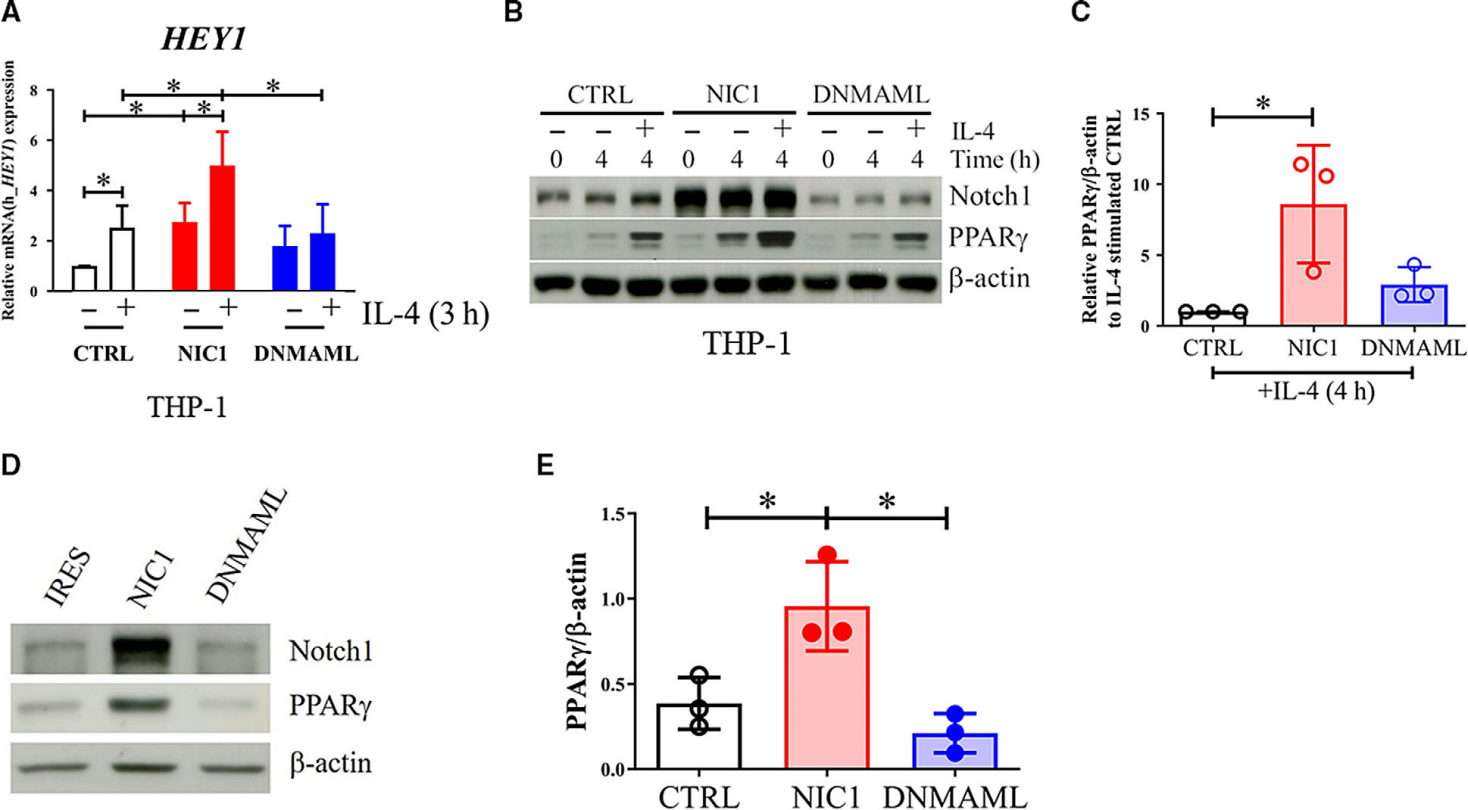
Effect of modifying Notch signaling on PPARγ expression in M(IL‐4). (A, B) Control, NIC1‐, or DNMAML‐overexpressing THP‐1‐derived macrophages were stimulated with IL‐4 (20 ng·mL^−1^) for indicated time. *HEY1* mRNA expression was determined by qPCR. Notch1 and PPARγ were detected by western blotting. * indicates statistically significant differences at *P* < 0.05 by two‐way ANOVA. (C) The densitometric intensity of PPARγ was normalized with β‐actin and compared to that of the IL‐4‐stimulated control. * indicates statistically significant differences at *P* < 0.05 by unpaired *t*‐test. (D) Control, NIC1‐, or DNMAML‐overexpressing THP‐1 at the monocytic stage was subjected to western blot to detect Notch1 and PPARγ. (E) The densitometric intensity of PPARγ was normalized with β‐actin. * indicates statistically significant differences at *P* < 0.05 by unpaired *t*‐test. The error bars represent mean ± SD.

To investigate the role Notch signaling plays in M(IL‐4), the THP‐1 cell line was subjected to retroviral transduction to overexpress NIC1 or the dominant negative form of MAML (DNMAML) to either increase or inhibit Notch signaling, respectively [33]. NIC1‐overexpressing THP‐1 cells showed increased *HEY1* mRNA with or without IL‐4 stimulation, while DNMAML overexpression resulted in the failure to upregulate *HEY1* mRNA upon IL‐4 activation (Fig. 3A). Therefore, NIC1 and DNMAML overexpression in the THP‐1 cell line displayed the phenotypes of hyperactivation and hypoactivation of the Notch signaling, respectively.

**Fig. 4:**
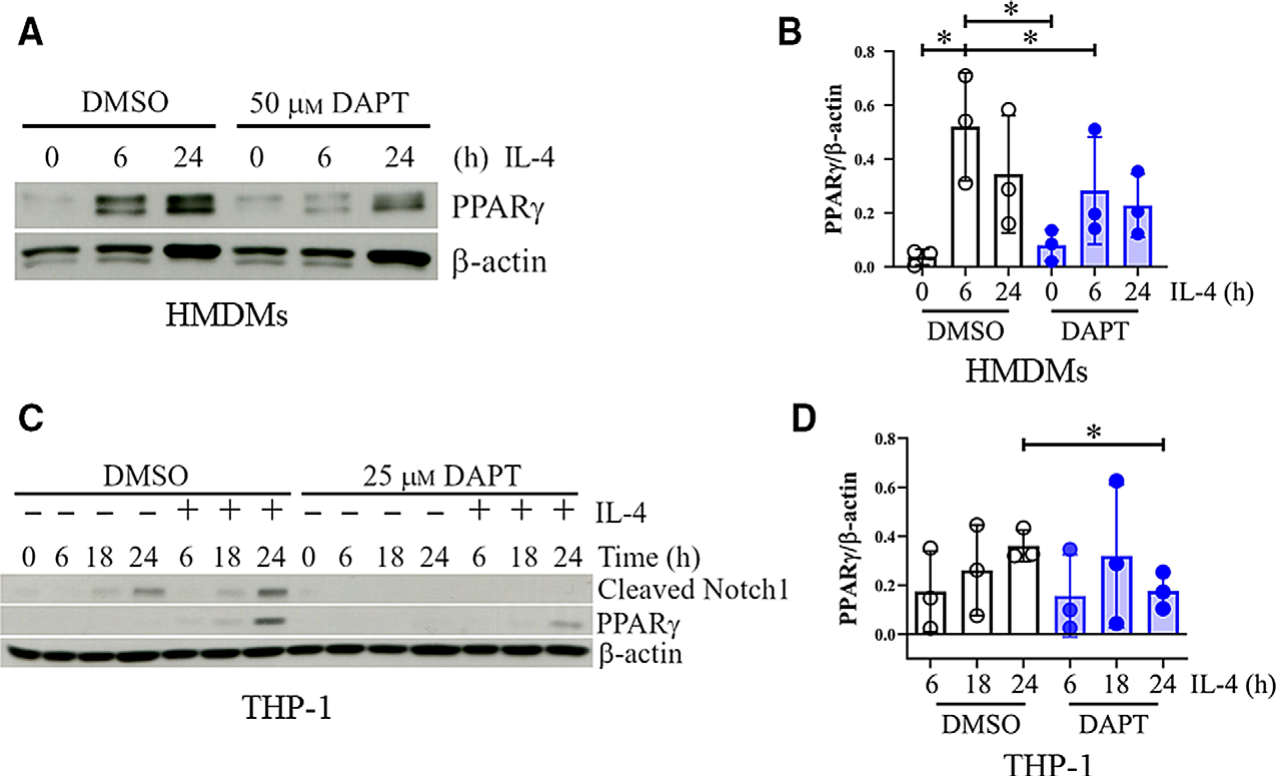
Effect of Notch signaling inhibitor on PPARγ expression in M(IL‐4). (A, B) HMDMs were pretreated with DAPT (50 μm), or (C, D) THP‐1‐derived macrophages were pretreated with DAPT (25 µm) before stimulation with IL‐4 (20 ng·mL^−1^) for indicated time. PPARγ protein expression was examined by western blotting. * indicates statistically significant differences at *P* < 0.05 by paired *t*‐test. (B, D) The densitometric intensity of PPARγ was normalized with β‐actin. The result is representative of three independent healthy donors or experiments. * indicates statistically significant differences at *P* < 0.05 by unpaired *t*‐test. The error bars represent mean ± SD.

**Fig. 5:**
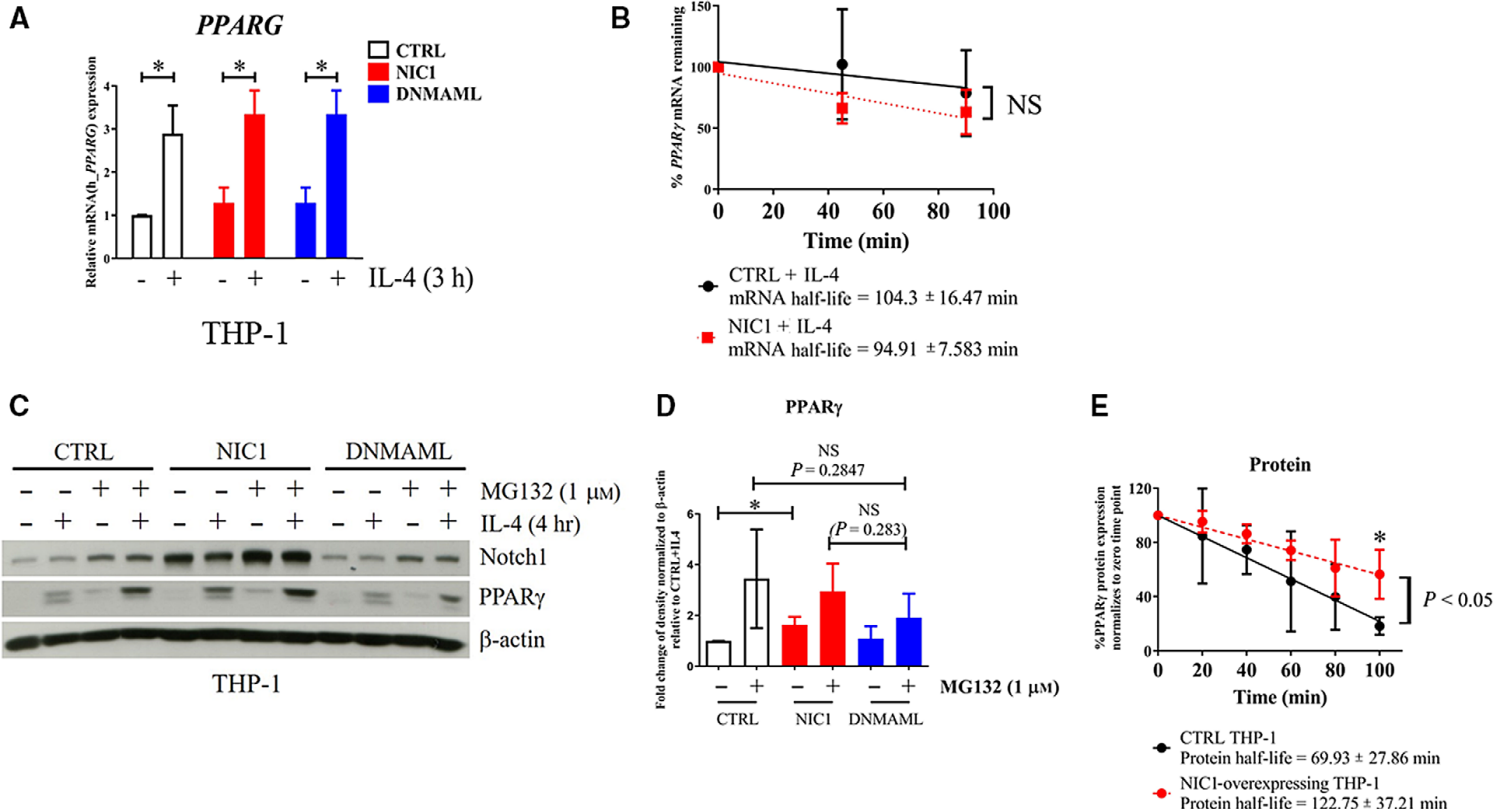
Stabilizing PPARγ by Notch signaling via preventing proteasomal degradation. (A) Control, NIC1‐, or DNMAML‐overexpressing THP‐1‐derived macrophages were stimulated with IL‐4 (20 ng·mL^−1^) for 3 h. Relative *PPARG* mRNA levels were determined by qPCR. * indicates statistically significant differences at *P* < 0.05 by two‐way ANOVA. (B) Control or NIC1‐overexpressing THP‐1 cells were stimulated with IL‐4 (20 ng·mL^−1^) for 3 h and subsequently treated with ActD (1 μg·mL^−1^) for 0, 45, and 90 min. *PPARG* mRNA expression was determined by qPCR. mRNA half‐life was calculated as described in the Materials and methods section. NS = no statistical significance. (C) Control, NIC1‐, or DNMAML‐overexpressing THP‐1‐derived macrophages were pretreated with MG132 (1 μm) for 1 h and subsequently stimulated with IL‐4 (20 ng·mL^−1^) for 4 h. Notch1 and PPARγ were detected by western blotting. (D) The relative densitometric intensity of PPARγ was normalized with β‐actin and compared to that of IL‐4‐stimulated control. (E) The decay graph of PPARγ protein expression in IL‐4‐activated control or NIC1‐overexpressing cells. PPARγ protein expression was normalized to that of β‐actin. The normalized expression was calculated as % PPARγ expression relative to 0 min of CHX treatment. The experiments were performed in three independent experiments. * indicates statistically significant differences at *P* < 0.05 by unpaired *t*‐test. The error bars represent mean ± SD.

Next, we investigated the effects of modulating Notch signaling on the level of PPARγ in these cells. PPARγ expression was increased in the IL‐4‐stimulated NIC1‐overexpressing THP‐1‐derived macrophages, compared with that in the control, while DNMAML‐overexpressing cells did not affect the PPARγ levels (Fig. 3B,C). To exclude the possibility that NIC1 might modulate PMA signaling and caused increasing of PPARγ, PPARγ protein expression was directly investigated in NIC1‐ or DNMAML‐overexpressing THP‐1 without PMA treatment. Similar result with the PMA treatment was obtained as NIC1 alone is sufficient to increase the level of PPARγ (Fig. 3D,E). Consistent with this finding, the DAPT‐pretreated HMDMs (Fig. 4A,B) or THP‐1‐derived macrophages (Fig. 4C,D) decreased PPARγ expression upon IL‐4 treatment. Spontaneously increased cleaved Notch1 in THP‐1 after 24 h was consistently observed with or without DMSO treatment. This increase may be the result of PMA‐induced macrophage differentiation of THP‐1 cell line. This observed phenomenon was much more delayed than IL‐4‐induced cleaved Notch1. When the *PPARG* mRNA was examined, NIC1 or DNMAML expression did not have any effect on the level of *PPARG* transcription in THP‐1‐derived macrophages (Fig. 5A). Taken together, these results indicated that Notch signaling enhanced PPARγ protein expression without any effect on its mRNA.

### Notch signaling regulates proteasomal degradation of PPARγ

Because interfering with Notch signaling did not alter the mRNA level of *PPARG*, we next examined the effect of Notch signaling on *PPARG* mRNA stability by measuring *PPARG* mRNA half‐life. As shown in Fig. 5B, control and NIC1‐overexpressing THP‐1‐derived macrophages exhibited a similar half‐life of *PPARG* mRNA upon IL‐4 stimulation. Therefore, Notch signaling did not regulate *PPARG* transcription or its mRNA stability. Previous reports found that the stability of PPARγ was regulated by proteasomal degradation [17, 34]. We then asked whether Notch signaling regulates the stability of PPARγ by this mechanism. THP‐1‐derived macrophages were pretreated with MG132, a proteasome inhibitor, and subsequently stimulated with IL‐4. The results clearly demonstrated that PPARγ levels were increased in MG132‐treated IL‐4‐stimulated cells, indicating the involvement of proteasomal degradation (Fig. 5C,D). In the NIC1‐overexpressing THP‐1‐derived macrophages, treatment with MG132 did not further increase the level of PPARγ (Fig. 5C,D). This result strongly indicated that NIC1 overexpression stabilized PPARγ protein through the prevention of proteasomal degradation.

To confirm that NIC1 prolongs PPARγ protein stability, the protein half‐life was measured in the control and NIC1‐overexpressing cells. The results showed that IL‐4‐stimulated NIC1‐overexpressing THP‐1 cells significantly extended the half‐life of PPARγ compared to that of the control (Fig. 5E) (69.93 ± 27.86 min in the control vs. 122.75 ± 37.21 min in NIC1‐overexpressing cells). Collectively, Notch signaling delays the proteasome‐dependent degradation of PPARγ in M(IL‐4).

### Notch signaling cross talks with the AKT pathway to regulate PPARγ protein stability

**Fig. 6:**
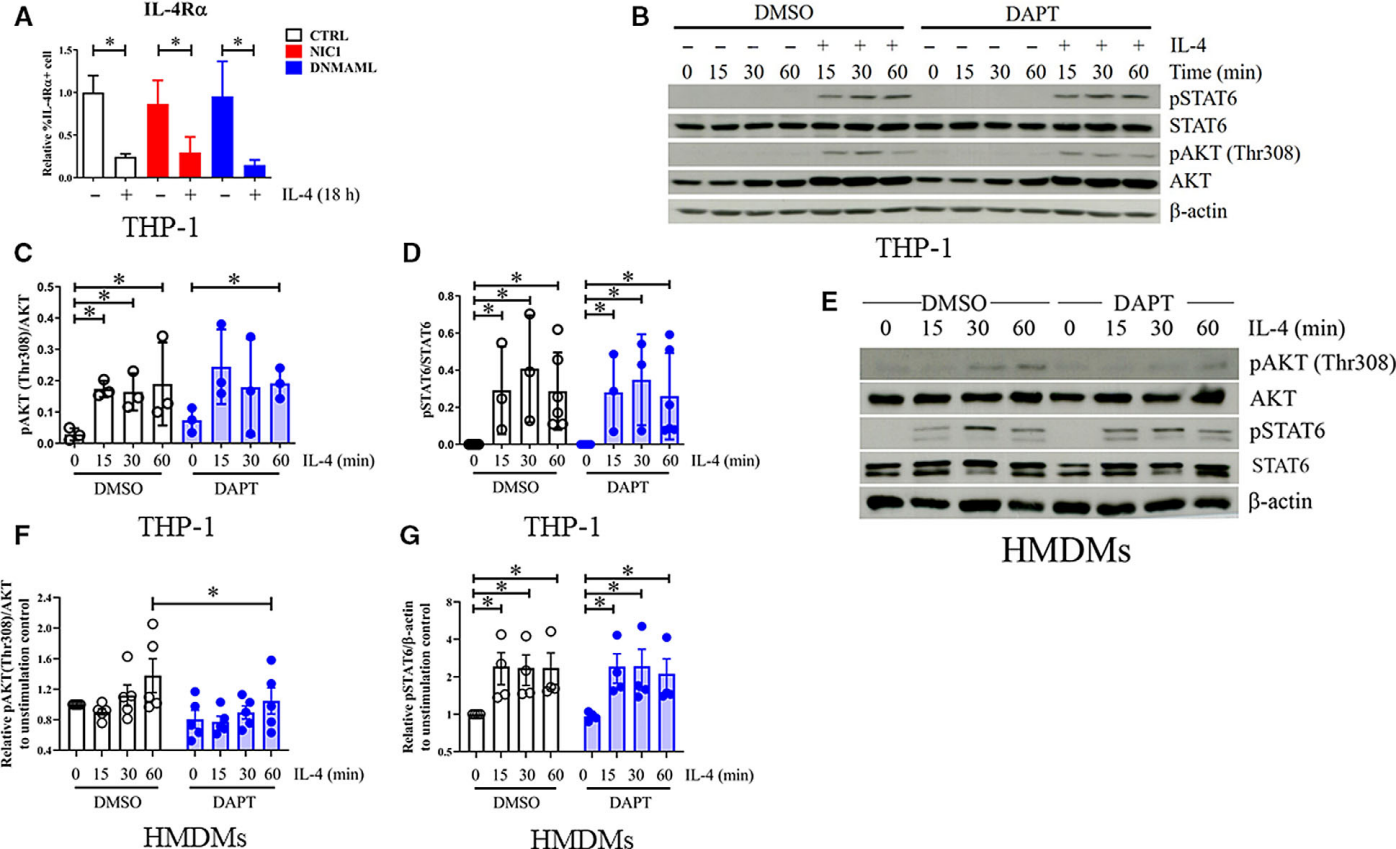
Regulation of IL‐4 downstream signaling by Notch signaling in M(IL‐4). (A) Control, NIC1‐, or DNMAML‐overexpressing THP‐1 cells were stimulated with IL‐4 (20 ng·mL^−1^) for the 18 h. IL‐4Rα expression was examined by flow cytometry. * indicates statistically significant differences at *P* < 0.05 by two‐way ANOVA. (B, D) THP‐1‐derived macrophages were pretreated with DAPT (25 µm), and (E–G) HMDMs were pretreated with DAPT (50 µm) before stimulation with IL‐4 (20 ng·mL^−1^) for indicated time. Phosphorylated and total form of AKT and STAT6 were examined by western blotting. (C) The densitometric intensity of pAKT (Ser473)/AKT and (D) pSTAT6/STAT6 was determined. (F) The relative densitometric intensity of pAKT/AKT and (G) pSTAT6/β‐actin was determined and compared to those of DMSO‐treated control at 0 min. The result is representative from five independent healthy donors or at least three independent experiments in THP‐1‐derived macrophages. * indicates statistically significant differences at *P* < 0.05 by unpaired *t*‐test. The error bars represent mean ± SD.

To explore how Notch signaling increases PPARγ in M(IL‐4), the level of IL‐4Rα was measured in the IL‐4‐activated THP‐1‐derived macrophages. As shown in Fig. 6A, IL‐4Rα decreased after IL‐4 stimulation whether Notch signaling is activated or inhibited, confirming the previous report, and NIC1 or DNMAML overexpression did not alter the IL‐4Rα expression level [35]. Next, the effect of Notch signaling on the downstream signaling of IL‐4/IL‐4R was investigated. DAPT‐pretreated THP‐1‐derived macrophages or HMDMs were stimulated with IL‐4, and the cell lysates were subjected to the detection of phosphorylation of STAT6 and AKT. DAPT‐pretreated THP‐1‐derived macrophages (Fig. 6B,C) exhibited delayed AKT (Thr 308) phosphorylation while DAPT‐pretreated HMDMs (Fig. 6E,F) decreased AKT (Thr308) phosphorylation. No effect of DAPT treatment was detected in IL‐4‐induced phosphorylation of STAT6 (Fig. 6D–G). This result suggests that Notch and AKT pathways cross talked in human M(IL‐4).

**Fig. 7:**
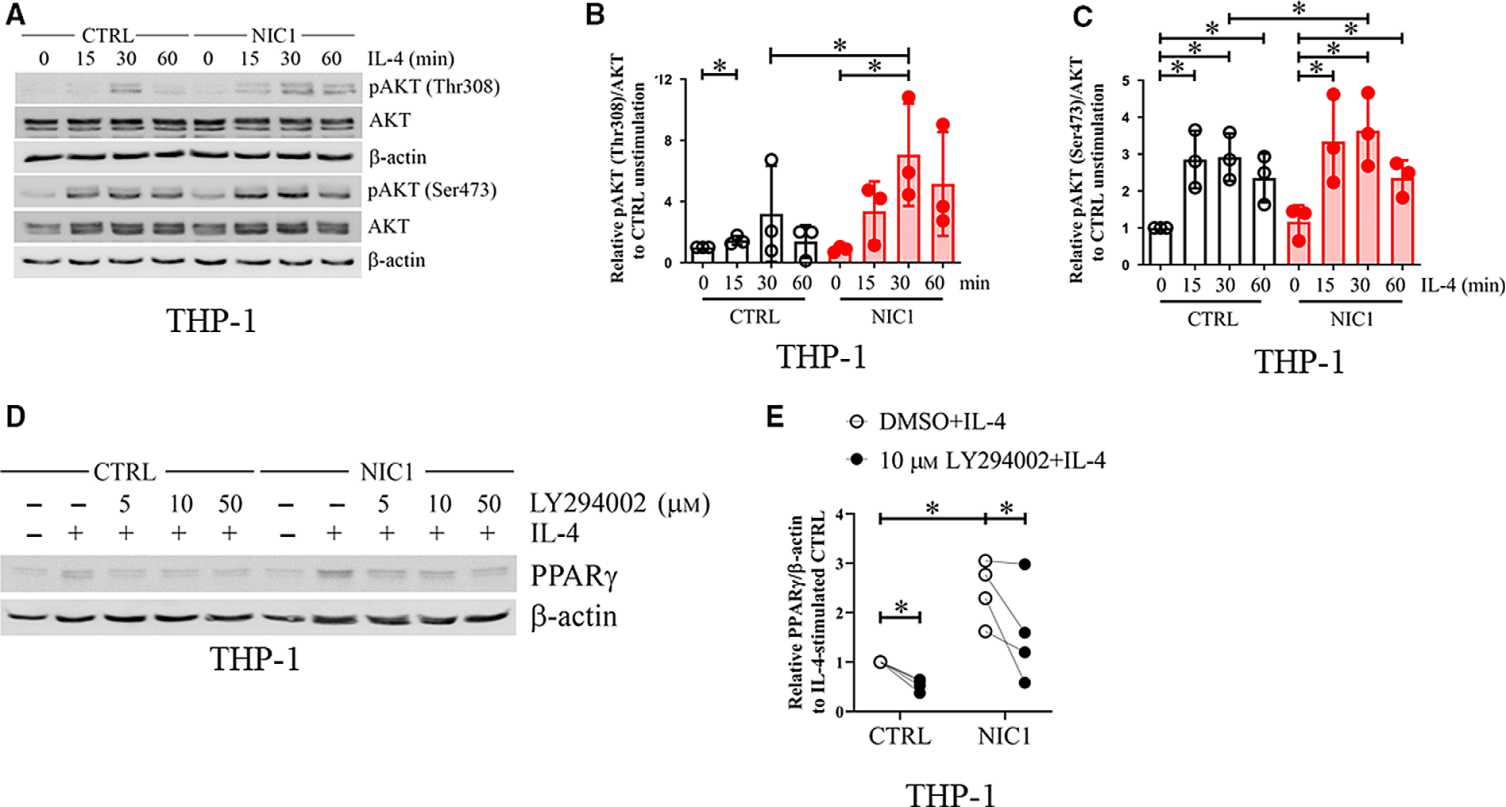
Cross talks between Notch signaling and AKT in M(IL‐4). (A) Control or NIC1‐overexpressing THP‐1‐derived macrophages were stimulated with IL‐4 (20 ng·mL^−1^) for indicated times. Phosphorylated AKT (Thr308 and Ser437) was detected by western blotting. (B) The relative densitometric intensity of pAKT (Thr308)/AKT and (C) pAKT (Ser473)/AKT was determined and compared to that of unstimulated control at 0 min. (D) Control or NIC1‐overexpressing THP‐1‐derived macrophages were pretreated with various concentrations of LY294002 or vehicle control DMSO for 1 h before stimulation with IL‐4 (20 ng·mL^−1^) for 4 h. PPARγ protein expression was examined by western blotting. (E) The relative densitometric intensity of PPARγ was normalized with β‐actin and compared to that of IL‐4‐stimulated control. * indicates statistically significant differences at *P* < 0.05 by *t*‐test. The error bars represent mean ± SD.

**Fig. 8:**
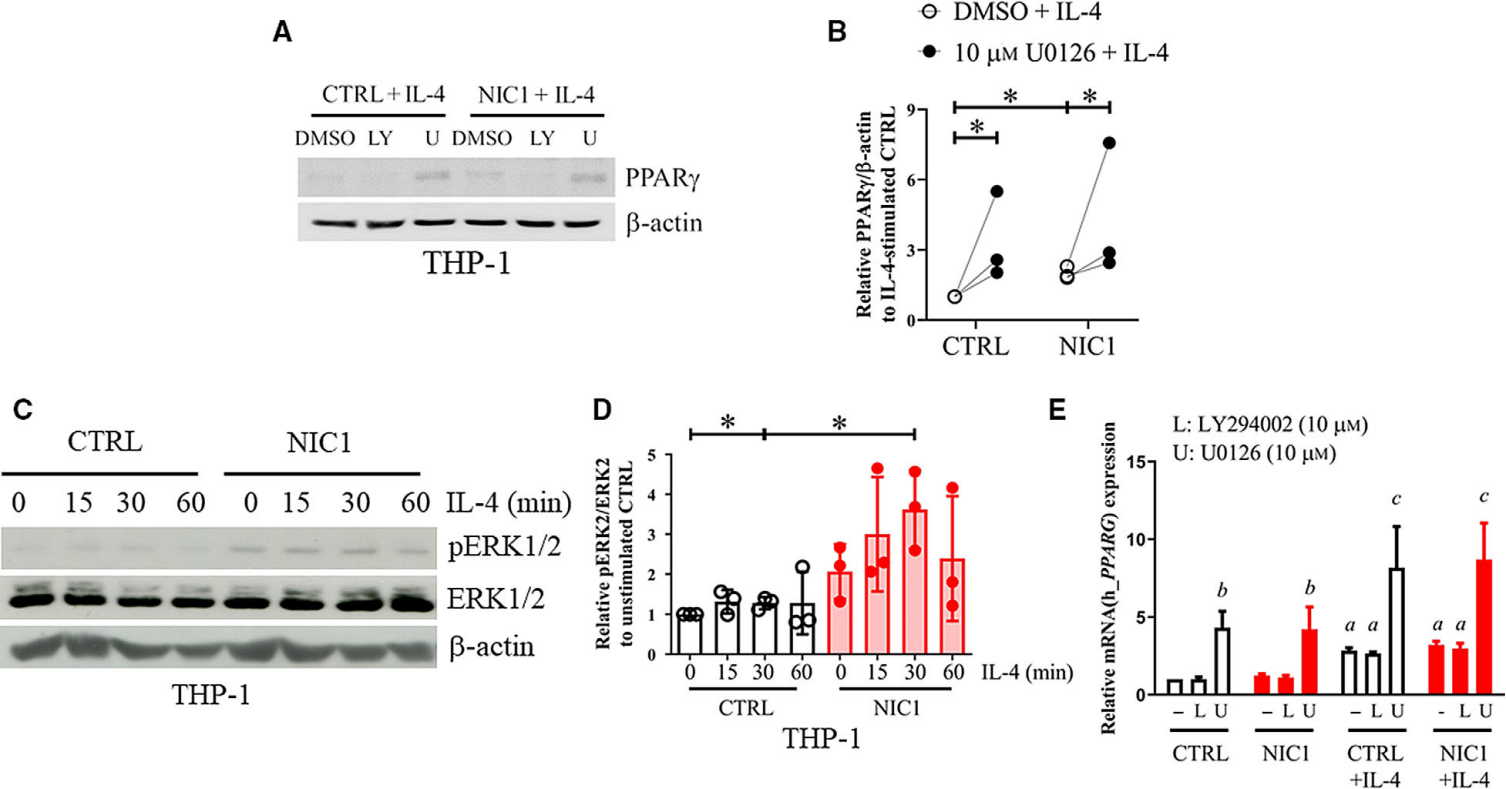
Cross talks between Notch signaling and AKT and ERK in M(IL‐4). (A) Control or NIC1‐overexpressing THP‐1‐derived macrophages were pretreated with LY294002 (10 μm, LY) or U0126 (10 μm, U) for 1 h before stimulation with IL‐4 (20 ng·mL^−1^) for 4 h. PPARγ protein expression was examined by western blotting. (B) The relative densitometric intensity of PPARγ was normalized with β‐actin and compared to that of IL‐4‐stimulated control. (C) Control or NIC1‐overexpressing THP‐1‐derived macrophages were stimulated with IL‐4 (20 ng·mL^−1^) for the indicated times. Phosphorylated ERK1/2 and total ERK1/2 were examined by western blotting. (D) The relative densitometric intensity of pERK2/ERK2 compared to that of unstimulated control at 0 min. * indicates statistically significant differences at *P* < 0.05 by *t*‐test. The error bars represent mean ± SD. (E) Control or NIC1‐overexpressing THP‐1‐derived macrophages were pretreated with vehicle control DMSO, LY294002 (10 μm), or U0126 (10 μm) or for 1 h before stimulation with IL‐4 (20 ng·mL^−1^) for 3 h. *PPARG* mRNA expression was determined by qPCR. The result was performed in three independent experiments. *a, b,* and *c* indicated statistically significant differences when compared with unstimulated condition, DMSO‐unstimulated condition, and IL‐4‐stimulated control (DMSO), respectively, with *P* < 0.05. The error bars represent mean ± SEM.

To investigate the effect of NIC1 overexpression on AKT phosphorylation, NIC1‐overexpressing THP‐1‐derived macrophages were treated with IL‐4 and the phosphorylation of AKT was detected. As shown in Fig. 7A–C, NIC1 overexpression significantly extended the duration of Thr308 phosphorylation while slightly increased the level of Ser473 phosphorylation. Next, the effect of specific inhibitors of AKT was tested on the level of PPARγ in M(IL‐4). The results showed that the PI3K/AKT inhibitor LY294002 reduced the PPARγ levels in NIC1‐overexpressing cells to the similar level as those of IL‐4‐stimulated THP‐1‐derived macrophages in a dose‐dependent manner (Fig. 7D,E). We also detected the effect of the ERK1/2 inhibitor U0126 where it increased the PPARγ levels (Fig. 8A,B). Consistent with this finding, NIC1 overexpression also increased the level of phosphorylated ERK1/2 (Fig. 8C,D). To examine the possibility of the inhibitor effects on mRNA transcription, the effects of LY294002 and U0126 on *PPARG* were examined at the mRNA level. As shown in Fig. 8E, the treatment with LY294002 did not alter the level of *PPARG* in all conditions tested, whereas the treatment with U0126 significantly increased *PPARG* levels. Together, these data suggest that Notch signaling cross talked with the PI3K/AKT and ERK pathways to negatively regulate *PPARG* transcription (via ERK) or positively regulate PPARγ protein stability (via PI3K/AKT).

### Notch signaling enhances CD36 expression and lipid accumulation in THP‐1 via PPARγ

**Fig. 9:**
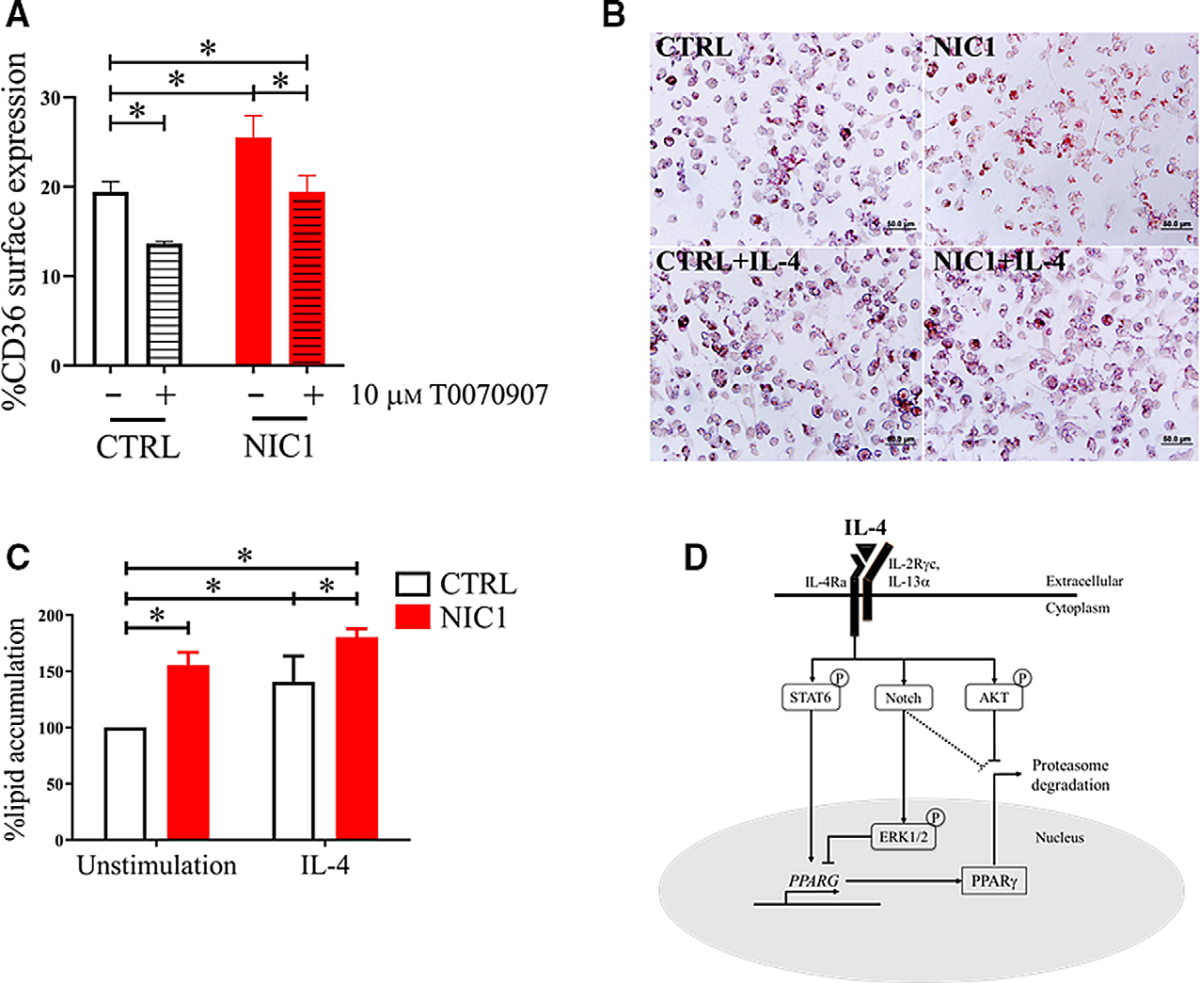
Enhancing CD36 expression and lipid accumulation by NIC1 overexpression in THP‐1 cell line. (A) Control or NIC1‐overexpressing THP‐1‐derived macrophages were treated with T0070907 (10 µm) for 18 h. Surface CD36 expression was detected by flow cytometry. (B, C) Control or NIC1‐overexpressing THP‐1‐derived macrophages were stained with Oil Red O (40×, scale bar 50 µm) (B), and the intracellular lipids were measured at 492 nm (C). The results represent three independent experiments. * indicates statistically significant differences when compared with the unstimulated condition with *P *< 0.05 by two‐way ANOVA. The error bars represent mean ± SD. (D) Schematic diagram showing the cross talk between Notch signaling, AKT, and ERK in M(IL‐4).

PPARγ regulates various key features of M(IL‐4) and IL‐4‐mediated induction both PPARγ and 12/15‐lipoxygenase that can generate PPARγ endogenous ligand, leading to PPARγ activation and upregulation of PPARγ target genes such as *CD36* [36]. Therefore, the impact of Notch signaling was examined on CD36 expression and lipid accumulation. As expected, the flow cytometry result showed that NIC1 overexpression alone is sufficient for increasing surface expression CD36 protein level (Fig. 9A). We further tested whether NIC1‐mediated upregulation of CD36 is mediated through PPARγ by using PPARγ‐specific antagonist, T0070907. As shown in Fig. 9A, T0070907 treatment significantly decreased CD36 protein level (Fig. 9A) suggests that NIC1 increases CD36 expression in PPARγ‐dependent manner. In our system, we could not detect IL‐4‐induced CD36 upregulation in THP‐1‐derived macrophages during the duration tested in this study and this is consistent with previous report [37].

Next, intracellular lipid accumulation in CTRL‐ or NIC1‐overexpressing THP‐1‐derived macrophages was investigated by Oil Red O staining. NIC1‐overexpressing THP‐1 cell line spontaneously accumulated lipids, and the level was increased upon IL‐4 stimulation (Fig. 9B,C). Therefore, Notch signaling in M(IL‐4) enhances the M(IL‐4) biological functions of lipid accumulation. The proposed cross talk between Notch signaling, AKT/ERK, and PPARγ is illustrated in Fig. 9D (see Discussion).

## Discussion

In this study, we uncovered that Notch signaling increases the stability of PPARγ and thus its biological functions in M(IL‐4). This result is consistent with the reported involvement of Notch signaling in pro‐healing macrophages [27, 28]. This finding argues against the current model that Notch signaling is pro‐inflammatory in macrophages such as in M(LPS) [22, 25]. LPS‐stimulated NIC1‐overexpressing THP‐1 cell line did not significantly increased TNFα expression compared to that of the control THP‐1 cell line (data not shown). It indicates that Notch signaling in macrophages may not function as an M1/M2 determining factor, but may rather operate in a context‐dependent manner so that it can enforce the primary stimulating signaling in macrophages, being either pro‐inflammatory (LPS) or pro‐healing (IL‐4).

The Notch receptor/ligand profiles in M(IL‐4) of both HMDMs and THP‐derived macrophages revealed that JAGGED1/2 are predominantly upregulated upon IL‐4 stimulation. Indeed, blocking JAGGED1 reduced the upregulation of one of target genes of the Notch signaling HEY1, indicating that JAGGED1 is the main ligand for Notch activation in M(IL‐4). We could not exclude the possibility of the involvement of other ligands in IL‐4‐stimulated human macrophages. Previously, Foldi *et al*. [38] reported that TLR stimulation induced Jagged1 upregulation in murine macrophages.

How does Notch regulate PPARγ stability in M(IL‐4)? We reported here that by cross talk with the PI3K/AKT pathway, Notch signaling prevents the proteasomal degradation of PPARγ without affecting its mRNA transcriptional level or its stability. Similar observation was reported on the role of STAT6 in facilitating PPARγ functions in IL‐4‐stimulated bone marrow‐derived macrophages without an impact on transcription or stability of its mRNA [39]. Notch signaling has been reported to closely cross‐regulate with the PI3K/AKT pathway in cancers, especially in T‐cell acute lymphoblastic leukemia (T‐ALL) [40].

The protein stability of PPARγ is regulated mainly by proteasomes in adipocytes, and several E3 ubiquitin ligases are reported but proteasomal regulations of PPARγ have not been reported in macrophages [41]. In adipocytes, E3 ubiquitin ligases function to either stabilize or facilitate proteasomal degradation of PPARγ. Seven in absentia homolog 2 (SIAH2) [42]and makorin ring finger protein 1 (MKRN1) [43] facilitate PPARγ degradation, while the tripartite motif protein 23 (TRIM23) regulates PPARγ ubiquitination to stabilize it [44]. Furthermore, an E3 ubiquitin ligase NEDD4 is reported to stabilize PPARγ by preventing it from proteasomal degradation in 3T3‐L1 cells [41]. We have preliminarily examined the expression of these genes in our system but unable to validate that these E3 ubiquitin ligases play any role in M(IL‐4). The identification of E3 ubiquitin ligase that are under regulation of Notch/AKT needs further investigation that will shed the light on how Notch and AKT cross talk that affects PPARγ stability in M(IL‐4).

As depicted in Fig. 9D, we propose that in M(IL‐4), the activation of Notch signaling results in PI3K/AKT activation and prevents the proteasome‐dependent degradation of PPARγ. This model proposes that Notch signaling is a facilitator of IL‐4‐induced polarization of macrophages.

In contrast, Notch signaling alone is sufficient to activate the ERK1/2 pathway that negatively regulates the transcription of *PPARG*. Notch1 activation has been reported to increase ERK1/2 expression in HER2^+^ breast cancer by suppressing PTEN expression [45]. In T‐ALL, the Notch target gene *HES1* negatively regulates PTEN expression, which is a tumor suppressor and negative regulator of the PI3K/AKT pathway [46]. However, in our study, NIC1 overexpression in THP‐1 did not alter PTEN protein expression, indicating that PTEN is not involved in the cross talk with AKT/ERK1/2 (data not shown). Therefore, Notch signaling in M(IL‐4) functions in dual modes in both negative and positive ways to regulate the PPARγ levels, possibly for the optimal levels of PPARγ protein, and perturbing this balance may lead to the dysregulated expression of PPARγ and its regulatory gene network.

CD36 is a transmembrane receptor for oxidized low‐density lipoprotein which functions in macrophage/foam cell formation. CD36 expression is transcriptionally regulated by PPARγ. [47]. IL‐4 stimulation of macrophages results in PPARγ expression and, as a result, increases lipid accumulation via CD36. Our results by NIC1 overexpression confirmed the function of PPARγ in lipid accumulation. We have performed efferocytosis using apoptotic thymocytes in DAPT‐treated and control THP‐1 cell line but did not observe any difference in this function (data not shown). Apoptotic cell alone was reported to increase efferocytic activity via the induction of PPARγ and CD36 in bleomycin induced lung injury mouse model [36]. Therefore, the regulation of Notch signaling to PPARγ and lipid accumulation may require additional signal(s) besides IL‐4 and CD36 for this biological function of M(IL‐4).

Both PPARγ and Notch play important roles in regulating cellular metabolisms in other cell types, including macrophages [48]. Whether they also regulate M(IL‐4) metabolism warrants further investigation as recent evidences suggests that M(IL‐4) utilizes a unique metabolic pathway that differs from that of M(LPS) [49].

In conclusion, Notch signaling plays both negative and positive roles in regulating mRNA and protein of PPARγ through the ERK and AKT pathways in human macrophages activated by IL‐4. Manipulating macrophage functions during polarization may need to take the Notch signaling into consideration for optimal outcome.

## Conflict of interest

The authors declare no conflict of interest.

## Author contributions

NS contributed to design of the work, acquisition of data, analysis and interpretation of data, and drafting of the article. PK contributed in experimental settings and data acquisition. TP contributed to conception and design of the work, analysis and interpretation of the data, funding acquisition, drafting, and revision of the article.
